# Sugar Swing After Surgery: Hyperinsulinemic Hypoglycemia With Possible Nesidioblastosis After Roux-en-Y Gastric Bypass Surgery

**DOI:** 10.7759/cureus.47349

**Published:** 2023-10-19

**Authors:** Hiwot T Workneh, Bijal Mehta, Anjali Grover

**Affiliations:** 1 Internal Medicine, Hackensack Meridian Mountainside Medical Center, Montclair, USA; 2 Endocrinology, Diabetes and Metabolism, Hackensack Meridian Mountainside Medical Center, Montclair, USA

**Keywords:** bariatric surgery, nesidioblastosis, gastric bypass, hypoglycemia, niph

## Abstract

Bariatric surgery is a procedure performed to achieve weight loss and manage obesity. However, it can result in various complications including post-surgical hypoglycemia. Nesidioblastosis is a rare hypoglycemic syndrome marked by diffuse hyperplasia of pancreatic β cells with distinct histologic features. Recent case reports have indicated an association of nesidioblastosis with certain bariatric procedures, often specifically linked to Roux-en-Y gastric bypass (RYGB) surgery.

In this case report, we describe a 78-year-old male with a complex medical history who presented with altered mental status and severe hypoglycemia (13 mg/dL), despite having no history of diabetes or use of hypoglycemic medications. The patient's clinical condition improved after receiving a 50% intravenous dextrose injection and subsequently placed on a 10% dextrose infusion. Adrenal insufficiency was ruled out with normal cortisol level, and tests for β-hydroxybutyrate, dehydroepiandrosterone (DHEA) sulfate, and hypoglycemia panels were all negative. However, further investigations were significant for elevated serum insulin, C-peptide, and proinsulin levels. The patient then underwent an abdominal computed tomography (CT) scan, which revealed a grossly normal liver, spleen, pancreas, and adrenal glands, along with evidence of prior gastric bypass surgery. Further evaluation confirmed a history of Roux-en-Y gastric bypass surgery, which was performed to address morbid obesity and obstructive sleep apnea. Following the procedure, the patient began experiencing hypoglycemic episodes. Subsequently, the patient was diagnosed with hyperinsulinemic hypoglycemia with possible nesidioblastosis. This diagnosis was made based on severe recurrent postprandial hypoglycemia, accompanied by elevated endogenous insulin production, and a pancreas that appeared grossly normal on imaging. The patient was treated with acarbose to prevent carbohydrate-driven blood sugar and insulin spikes, octreotide to inhibit insulin secretion, and dietary guidance to avoid high glycemic index foods. This case emphasizes the potential link between bariatric surgeries and metabolic disturbances, underscoring the importance of identifying uncommon hypoglycemic syndromes.

## Introduction

Bariatric surgery is a weight loss and obesity management procedure [1], but it can lead to several metabolic and endocrine complications, including postoperative hypoglycemia [2]. These complications are mainly associated with the Roux-en-Y gastric bypass (RYGB) procedure [2,3]. Recent case reports have shown an association of this procedure with hyperinsulinemic hypoglycemia mainly resulting from nesidioblastosis [4-7]. Nesidioblastosis is a rare hypoglycemic syndrome characterized by excessive growth of pancreatic β cells with distinct histologic features [5,7]. This condition is a rare occurrence, observed in approximately 0.1%-0.3% of cases [4,5]. Here, we present a case of a 78-year-old male with a history of gastric bypass surgery who presented with severe hypoglycemia secondary to endogenous hyperinsulinemia with a possible diagnosis of nesidioblastosis.

## Case presentation

A 78-year-old male patient with a medical history of dementia, hypothyroidism, hypertension, and a controlled seizure disorder (managed with divalproex) along with chronic kidney disease presented with altered mental status. On examination, there was no acute distress, and vital signs were stable. However, significant confusion and disorientation were observed without any gross motor or sensory deficits. Point-of-care testing revealed severe hypoglycemia, with a blood glucose level of 13 mg/dL. Notably, the patient had no history of diabetes or use of any hypoglycemic agents.

**Figure 1:**
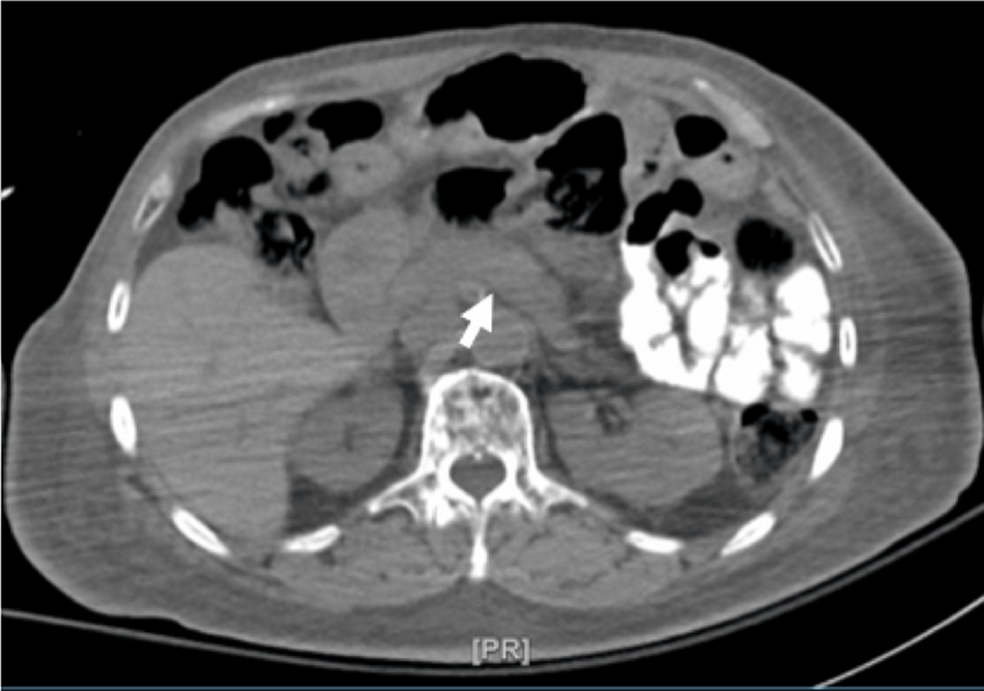
Axial CT scan of the abdomen showing normal appearance of the pancreas (white arrow) CT: computed tomography

**Figure 2:**
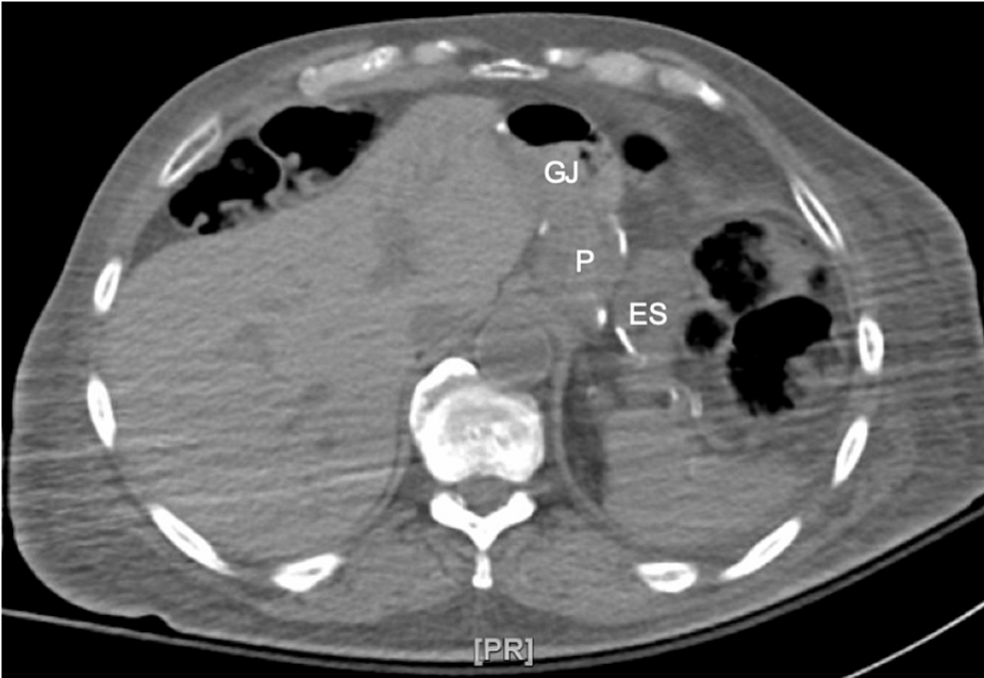
Axial CT scan of the abdomen showing a gastric pouch (P) laterally separated from the excluded stomach (ES), with a gastrojejunal (GJ) anastomosis anteriorly connected to the gastric pouch CT: computed tomography, P: gastric pouch, ES: excluded stomach, GJ: gastrojejunal

A computed tomography (CT) scan of the head showed no acute abnormalities. Further laboratory results indicated a normal thyroid-stimulating hormone (TSH) level of 2.25 µIU/mL and a cortisol level measuring 13.2 µg/dL. Hemoglobin A1c was found to be 4%, reflecting a lower-normal value. These initial results provided insight into the patient's metabolic and endocrine status, and the patient received a 50% intravenous dextrose injection, which improved the overall clinical state. However, due to recurrent severe hypoglycemia, the patient was admitted to the progressive care unit after initiating a continuous 10% dextrose infusion. Various potential causes were taken into consideration, including hypoglycemia induced by different medications including insulin and various hypoglycemic agents, insulinoma, liver abnormalities, autoimmune conditions, and primary hormonal deficiencies. Adrenal insufficiency was ruled out with normal cortisol level, and tests for β-hydroxybutyrate, dehydroepiandrosterone (DHEA) sulfate, and hypoglycemia panel (plasma levels of acetohexamide, chlorpropamide, tolazamide, tolbutamide, glimepiride, glipizide, glyburide, nateglinide, and repaglinide) were all negative. However, further investigations were significant for elevated serum insulin (92.2 µIU/mL), C-peptide (10.5 ng/mL), and proinsulin (10.6 pmol/L) levels. CT imaging of the abdomen revealed grossly normal liver, spleen, pancreas, and adrenal glands (Figure 1), with evidence of previous gastric bypass surgery (Figure 2).

Upon further evaluation, the patient's history of Roux-en-Y gastric bypass surgery, which was performed approximately 10 years ago, was confirmed. The patient had a history of morbid obesity with a body mass index (BMI) exceeding 45 kg/m², which had led to obstructive sleep apnea (OSA). The patient achieved substantial weight loss following the surgery but later began to encounter frequent and severe hypoglycemic episodes. Some of the episodes were severe enough to necessitate hospitalization. Subsequently, based on the evidence of severe recurrent postprandial hypoglycemia and the normal radiographic appearance of the pancreas in the context of prior bariatric surgery, the patient received a diagnosis of hyperinsulinemic hypoglycemia with possible nesidioblastosis. Treatment included acarbose for recurrent hypoglycemia management, octreotide to inhibit insulin secretion, dietary guidance to avoid high glycemic index foods, and replacement of divalproex with levetiracetam to reduce the risk of hypoglycemia. This case highlights the potential association between bariatric surgeries and metabolic disturbances, underscoring the significance of identifying rare hypoglycemic syndromes.

## Discussion

Bariatric surgery, commonly referred to as weight loss surgery, is a surgical intervention aimed to facilitate substantial weight loss and improve the overall well-being of individuals with severe obesity [1]. The two primary surgical methods widely recognized as standard practices for weight loss are laparoscopic sleeve gastrectomy and laparoscopic Roux-en-Y gastric bypass surgeries [3]. These procedures have remarkable success in reducing the incidence of diabetes in obese individuals and reducing mortality rates in those with severe obesity [1]. While bariatric surgery is generally considered safe and effective for weight loss, it also carries potential risks and complications related to metabolic abnormalities [2]. These interventions may lead to nutritional deficiencies and dumping syndrome as a consequence of interference with the digestive system [2]. Additionally, specific bariatric procedures are known to be linked with infrequent hypoglycemic episodes arising from abnormalities in islet cells, a condition often referred to as nesidioblastosis [2,8].

Nesidioblastosis is a rare medical condition characterized by endogenous hyperinsulinemic hypoglycemia. It results from excessive insulin production due to the hyperplasia of islet cells leading to an excess of β cell mass [9]. While this condition typically occurs in infants and children, it can rarely occur in adults. When it occurs in adults, it is often associated with prior Roux-en-Y gastric bypass surgery and typically presents with symptoms of severe hypoglycemia [9]. Diagnosing nesidioblastosis requires ruling out alternative causes such as insulinoma, factitious hyperinsulinemia, and autoimmune disorders [9]. Treatment might necessitate surgical measures such as total or subtotal pancreatectomy for curative measures. It is worth emphasizing that nesidioblastosis differs from insulinoma, a pancreatic tumor that can also lead to excessive insulin production [7,10]. In this case report, the patient had a history of a procedure that involved creating a small stomach pouch in the upper part of the stomach and redirecting a portion of the small intestine to this pouch. This surgical alteration can potentially lead to rapid food passage and a quick release of insulin, resulting in post-meal hypoglycemia [11]. Additionally, the increase in glucagon-like peptide-1 (GLP-1) due to these physiological and anatomical changes in the digestive system and food processing can also contribute to hypoglycemia by stimulating the growth of pancreatic β cells and enhancing insulin production, resulting in exaggerated insulin spikes [5]. However, it is important to highlight that post-bariatric surgery hypoglycemia typically results from a combination of factors. These factors encompass chronic irregularities in glucose regulation, shifts in glucose processing, hormonal fluctuations, and modifications in the structure and function of β cells [9,12]. These intricate interactions are potentially shaped by persistent anatomical and metabolic changes following the procedure [13]. In this case report, we documented an instance of hyperinsulinemic hypoglycemia with possible nesidioblastosis associated with gastric bypass surgery.

## Conclusions

Bariatric surgery, a common approach for substantial weight loss in severe obesity, includes procedures such as Roux-en-Y gastric bypass. While generally effective, these surgeries carry risks, including metabolic complications and hormonal imbalances. Nesidioblastosis is a rare condition marked by overproduction of insulin due to β cell hyperplasia leading to hypoglycemia, which has been associated with specific bariatric procedures. This case highlights the complex relationship between Roux-en-Y gastric bypass surgery and subsequent recurrent severe hypoglycemia syndrome due to changes in pancreatic β cell mass and increased insulin production. Recognizing such rare hypoglycemic syndromes is crucial for accurate diagnosis and effective management, in the context of bariatric interventions.
